# Nanobiomotors of archaeal DNA repair machineries: current research status and application potential

**DOI:** 10.1186/2045-3701-4-32

**Published:** 2014-06-25

**Authors:** Wenyuan Han, Yulong Shen, Qunxin She

**Affiliations:** 1 State Key Laboratory of Microbial Technology, Shandong University, Jinan, People's Republic of China; 2 Archaeal Centre, Department of Biology, University of Copenhagen, Copenhagen Biocenter, Copenhagen, Denmark

**Keywords:** Archaea, Nanobiomotor, Helicase, RecA fold, DNA repair

## Abstract

Nanobiomotors perform various important functions in the cell, and they also emerge as potential vehicle for drug delivery. These proteins employ conserved ATPase domains to convert chemical energy to mechanical work and motion. Several archaeal nucleic acid nanobiomotors, such as DNA helicases that unwind double-stranded DNA molecules during DNA damage repair, have been characterized in details. XPB, XPD and Hjm are SF2 family helicases, each of which employs two ATPase domains for ATP binding and hydrolysis to drive DNA unwinding. They also carry additional specific domains for substrate binding and regulation. Another helicase, HerA, forms a hexameric ring that may act as a DNA-pumping enzyme at the end processing of double-stranded DNA breaks. Common for all these nanobiomotors is that they contain ATPase domain that adopts RecA fold structure. This structure is characteristic for RecA/RadA family proteins and has been studied in great details. Here we review the structural analyses of these archaeal nucleic acid biomotors and the molecular mechanisms of how ATP binding and hydrolysis promote the conformation change that drives mechanical motion. The application potential of archaeal nanobiomotors in drug delivery has been discussed.

## Introduction

Biomolecules exhibit good potential in nanomedicine since they can be used to deliver new potential drugs such as short interference RNAs (siRNAs). Currently, lipid nanoparticles represent the most advanced biomolecular system for delivering siRNAs with which therapeutic effects have been observed [1]. However, the usefulness of this type of nanoparticle is compromised by the finding that multiple cellular signaling effectors are required for initial cellular entry of lipid nanoparticle during which the internalized siRNAs have undergone exocytosis [2]. Conceivably, it is very important to investigate other biomolecules for their potential in safe and effective drug delivery.

Biomolecular motors (nanobiomotors) belong to an interesting class of biomolecules that exhibit a good potential in the biotechnological application. Nanobiomotors convert chemical energy into mechanical work and motion, a reaction essential for many cellular processes in living organisms, and they are widespread in every living organism on Earth regardless whether they belong to Bacteria, Archaea or Eukarya, collectively as the three domains of life. Organisms of the two former domains comprise of single cellular prokaryotes that often co-inhabit in environments whereas the latter includes higher eukaryotes such as human beings. Being prokaryotic, Archaea and Bacteria share some traits such as lacking any distinct nuclear membrane to separate their genetic materials from the cytoplasm and utilizing similar metabolic pathways. However, Archaea is only distantly related to Bacteria phylogenetically [3]. This is reflected by the conservation of information-processing machineries in Archaea and Eukarya, including molecular machineries of chromosome replication, DNA damage repair, gene transcription and protein translation [4,5].

Specifically, many archaeal DNA repair proteins have homologs in eukaryotes, including those involved in base excision repair (BER), nucleotide excision repair (NER) and homologous DNA recombination-mediated DNA repair (HRR) [4,6,7]. BER proteins are well conserved in all three domains of life and exhibit similar structures, but archaeal BER proteins still show higher sequence similarities to the eukaryotic corresponding enzymes than to their bacterial counterparts. For the two general DNA repair pathways NER and HRR, archaeal and eukaryal proteins involved in, or implicated in, the processes, are of the same type, whereas the bacterial counterparts typically belong to another category.

Only some of the eukaryal NER proteins have a homolog in Archaea, including XPB and XPD helicases and, XPF and XPG/Fen-1 nucleases, because archaea also encode several lineage-specific NER proteins. For example, some archaeal XPBs interact with another archaea-specific nuclease Bax1, which is thought to be a functional homolog of eukaryal XPG nuclease. The archaeal XPG/Fen-1, on the other hand, is shown to be an essential enzyme implicated in the maturation of Okazaki fragments during chromosome replication [8]. Further, functional studies of archaeal NER enzymes have revealed interesting differences between archaeal and eukaryal XPB and XPD proteins. Whereas archaeal enzymes are dispensable for cell viability, their eukaryal counterparts have essential functions in gene transcription by RNA polymerase II [9-11].

Homologous recombination proteins conserved in Archaea and Eukarya include Rad50/Mre11 (MR) complex and the RadA/Rad51 recombinase. The former, together with some lineage-specific helicases and nucleases, is responsible for processing double-stranded DNA (dsDNA) ends into 3’-pertruding ends [12] to which the latter binds and mediates the strand invasion reaction in homologous recombination. Eukaryotic-specific helicases and nucleases involved in the processing of DNA ends include Sgs1 and Sae2, Exo1 in yeasts [13] and BLM and Dna2, Exo1 in humans [14], whereas archaeal-specific counterparts are nuclease NurA and helicase HerA [12]. Moreover, archaea and eukaryotes encode RadA/Rad51 paralogs, such as Rad55/57 in yeast, Rad51B/C/D, Xrcc2 and Xrcc3 in mammals, and RadB, RadC in Archaea, which facilitate homologous recombination by interacting with RadA/Rad51 recombinases [15-17].

After strand invasion, DNA synthesis at the heteroduplex region and subsequent ligation yield a Holliday junction intermediate, which needs to be resolved by a joint action of helicase and nuclease at a late stage of the homologous recombination pathway [18,19]. RecQ-like enzymes, including eukaryotic helicases WRN and BLM, are responsible for Holliday junction structure resolution and re-starting stalled DNA replication forks [20,21]. Archaea also encode one such homolog, either named Hjm (Holliday junction migration) or Hel308, which could perform the same functions [22,23].

A common task in NER and HRR is that the machineries need to open up double stranded DNA (dsDNA) at the very first step. As dsDNA has a very stable structure, nature has invented nucleic acid nanobiomotors, namely DNA helicases to do the job, and the process is powered by ATP hydrolysis. In addition to helicase motors, RecA family recombinases catalyze the strand exchange reaction in homologous recombination, representing another class of well-characterized nanobiomotors. This review will summarize the biochemical and structural analyses of the archaeal recombinases and helicases and their possible modes of action and discuss their possible application as nanomaterials.

## DNA repair helicases are nanobiomotors driven by ATP binding and hydrolysis

Helicases are ATPases that unwind DNA or RNA duplex. These molecular motors convert energy from ATP hydrolysis to motion that unwinds nucleic acids. Helicases fall into six superfamilies: The SF3 to SF6 family helicases form a ring structure, while SF1 and SF2 family helicases function as monomers [24]. Three known DNA repair helicases in Archaea fall into the SF2 family, including NER helicases XPB, XPD and RecQ-like helicase Hjm. These archaeal helicases contain two conserved ATPase domains each of which binds ATP, promoting conformational changes upon ATP hydrolysis. Other domains are not conserved in different types of SF2 enzymes and function in substrate binding and/or in regulating the helicase activity. Crystal structures are available for representatives of all three types of archaeal SF2 helicases [25-27].

The discovery that components of archaeal NER machinery are homologous to eukaryotic NER proteins has greatly facilitated the research of archaeal/eukaryotic NER enzymes, namely the NER helicases and nucleases. To date, several archaeal enzymes are well characterized including XPB, XPD, XPF and XPG/Fen1 as well as a few archaea-specific nucleases. The obtained results are incorporated into a model accounting for the putative archaeal NER process [28]: (1) the recognition of the damaged DNA by the single-stranded DNA binding protein (SSB), (2) SSB recruits other repair proteins to the damaged site via the C-terminal tail, (3) a helicase-nuclease complex formed by XPB and its partner Bax1 (bind to archaeal XPB) is recruited to one side of the damaged DNA whereas XPD and XPF may work at the other side. DNA damage recognition could also occur via archaeal XPB proteins since these proteins could employ the DNA damage recognition domain (DRD) to detect DNA damage.

**Figure 1:**
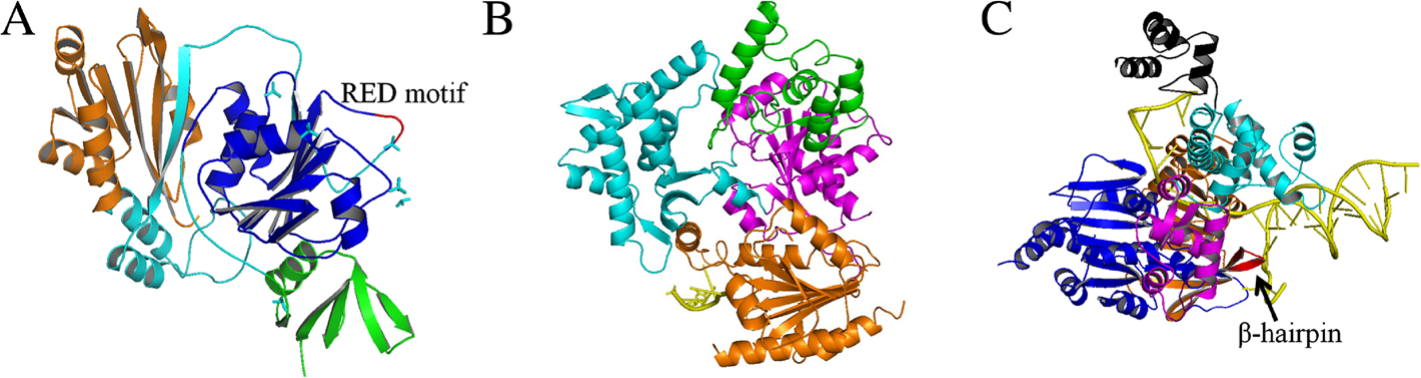
**(A) Structure of AfuXPB (PDB: 2FWR).** The DRD domain implicated in DNA damage recognition is colored green and the two ATPase motors are in blue and orange. The cyan part shows the flexible thrum motif inserted in the HD2 domain. The RED motif, which may function to open dsDNA, is shown in red. **(B)** Structure of TaXPD-DNA complex (PDB: 4A15). The ATPase motors are shown in magenta and orange. Two domains are inserted into HD1: the 4FeS domain (green) and Arch domain (cyan). DNA is shown in yellow. **(C)** Structure of AfuHel308-DNA complex (PDB: 2P6R). The ATPase motors are shown in blue and orange. The other three domains are in magenta, cyan and black, respectively. The putative site for duplex DNA melting, β-hairpin, is shown in red.

**Figure 2:**
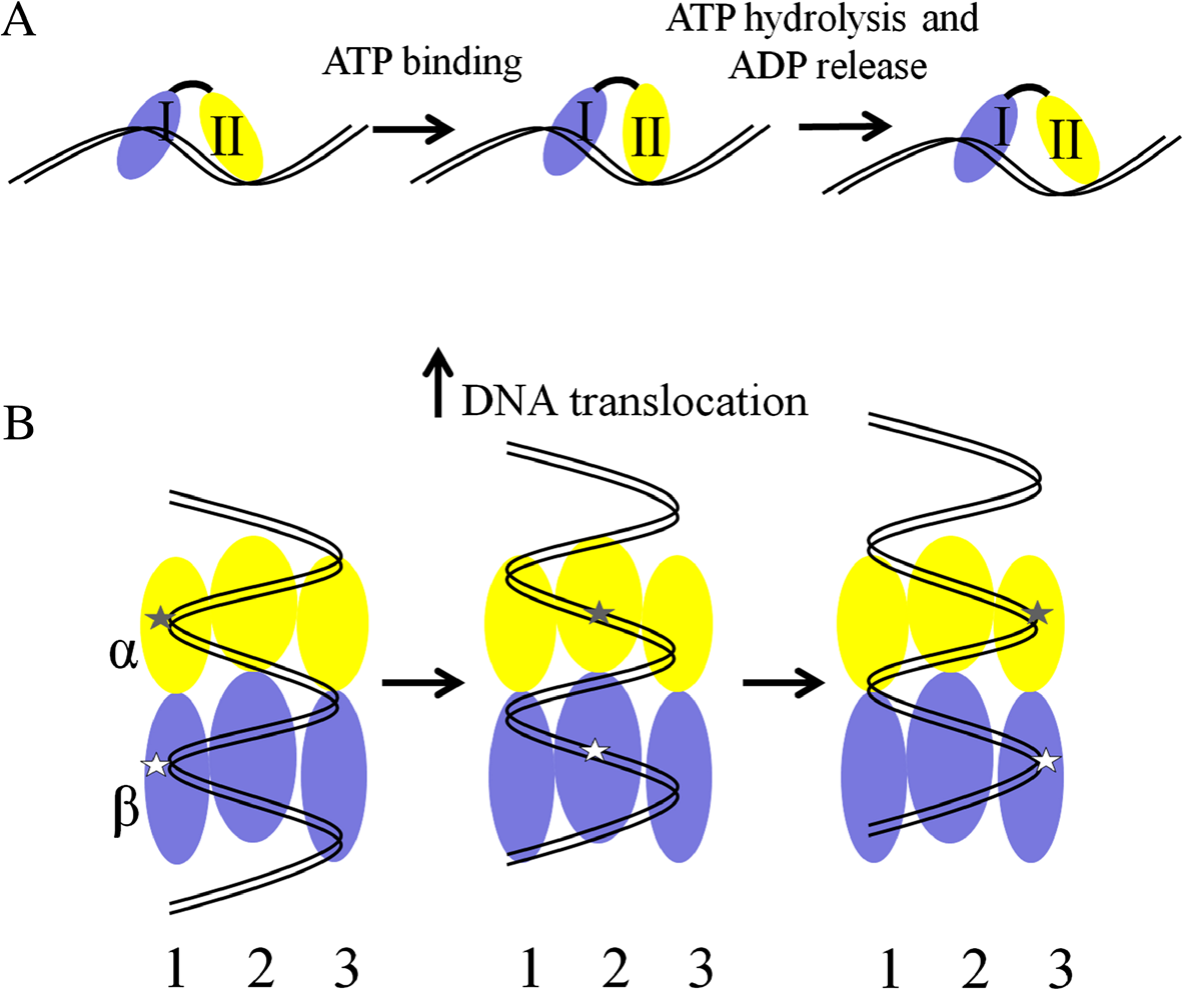
**(A) Rotation model by SF2 helicase (adapted from Buttner et al. **[34]**).** This type of nanobiomotor works as monomers and DNA unwinding occurs in two steps: (1) ATP binding leads to a structural change of the enzyme which pushes the two helicase domains closer. After ATP hydrolysis and ADP release, the protein conformation resets, resulting in a movement of the protein along DNA. **(B)**. Rotatory inchworm model by Ftsk (adapted from Massey et al. [46]). Each subunit moves along the DNA molecule in an inchworm manner as illustrated in 2A. Conformation change of the two DNA-binding sites in Subunit 1 (marked with stars) will lead to the interaction between DNA and the adjacent subunit (Subunit 2) due to the helical nature of DNA. Then, Subunit 2 completes its translocation upon ATP hydrolysis and ADP release, facilitating the interaction between Subunit 3 and the DNA target, and the cycle continues. Together, all subunits work sequentially with the rotation of the binding sites around the hexamer and the system proceeds like a rotatory inchworm. The arrow indicates the direction of DNA translocation.

Structure analysis of AfuXPB has revealed possible mechanism for DNA unwinding. The protein contains two canonical RecA-like helicase domains (HD1 and HD2) that are connected by a flexible hinge and DRD domain for DNA damage recognition [25]. Comparing to the structure of NS3 RNA helicase domain bound by a oligonucleotide ligand [29], the ligand-free AfuXPB is more open, suggesting that this helicase could use its substrate to regulate the conformational change, which will then lead to rotation of the protein on DNA substrate. The mechanism could be described as fowllowing. The helicase binds to DNA substrate via the DRD domain and promotes the rotation between two ATPase domains to form a closed XPB-DNA complex in which ATP is bound at the interface of the two ATPase domains. This conformation could mediate rotation since ATP hydrolysis and subsequent ADP release would lead to the change of relative positions of the two ATPase domains, driving the protein to move to the upstream of the double-stranded DNA [30]. Moreover, the rotation will also expel a conserved loop from HD2 between two DNA strands. This loop, named RED motif, has been shown to be important for the helicase activity and implicated in DNA unwinding (Figure 1A). Taken together, this indicates that XPB helicase employs a rotatory mechanism to move along DNA (Figure 2A) during which the RED motif opens dsDNA utilizing the energy from ATP hydrolysis.

The other NER helicase, XPD also carries ATPase domains (HD1 and HD2), processing a similar interface for ATP binding although unique accessory domains (4FeS and Arch domains) are inserted into the HD1 domain [26]. In the crystal structure of *Thermoplasma acidophilum* XPD (TaXPD) complexed with ssDNA substrate, the DNA segment inside the protein is gripped by a helix-loop-helix structure of HD2 (Figure 1B), which reveals a possible mechanism for XPD translocation polarity [31]. Unlike for XPB, the structure failed to reveal the unwinding site in the XPD helicase. While it is possible the site may be located within the 4FeS domain, this domain lacks a wedge-like DNA-binding site known as RED in XPB. It is worth pointing out that there is in fact a central puzzle in archaeal NER research: while in vitro studies have demonstrated helicase activity for all tested archaeal XPB and XPD enzymes [28], but none of the putative NER genes appear to be involved in DNA damage repair [10]. This suggests that the functions of archaeal putative NER proteins need to be carefully evaluated. Nevertheless, these nanobiomotors should have a function in DNA transactions in these unique organisms since these enzymes are well conserved across the entire Archaeal Domain.

Homologous recombination activity has been appreciated widely in maintaining genome integrity in all three domains of life. The process involves a Holliday junction that is resolved by a RecQ helicase that is conserved in Eukarya. An archaeal enzyme that may play the same role was first identified from *Pyrococcus furiosus* since its recombinant enzyme exhibited an ATP-dependent dissolution of DNA substrates with a Holliday junction [22]. Then, several archaeal RecQ enzymes were found to have the same activity, and they are the archaeal homologs of eukaryotic RecQ protein (designated either as Hjm or as Hel308). These archaeal enzymes show ca. 30% amino acid sequence identities among themselves [32] and are capable of unwinding DNA in 3’-5’ direction [22,32] albeit the *S. tokodaii* Hjm unwinds dsDNA substrates in both directions [23]. Genetic studies of archaeal Hjm proteins show that the gene is essential in *S. islandicus*, a hyperthermophilic crenarchaeon [33] but not in *T. kodakarensis*, a hypertherthermophilic euryarchaeon [9]. Since homologous recombination is essential in both organisms, this suggests there could be functional redundancies of Hjm in the latter.

Crystal structure has been determined for a few Hjm enzymes [34-36]. The *Archaeoglobus fulgidus* Hjm protein (AfuHel308) exhibits a five-domain structure, including two ATPase domains (domain 1 and 2) and three accessory domains. In an AfuHel308-DNA complex, domain 4, together with domain 1 and domain 3, forms a ring to hold 3’ tail ssDNA, while a β-hairpin structure from domain 2 wedges at the junction of ssDNA and dsDNA (Figure 1C). Interestingly, in the ATP-free state of AfuHel308, the two ATPase domains are separated by two intermediary bases. By contrast, in the ATP-bound state, the two ATPase domains form a closed conformation with only one intermediary base between them. Together, this provides a clue to the conformational change between the two states. During the ATP binding process, the γ-phosphate interacts with a conserved arginine residue from domain 2. The movement of domain 2 then pushes domain 4, and the conformational change releases the 3’ tail ssDNA across domain 1, finishing the translocation. Furthermore, the translocation will allow domain 2 to bind and melt more base pairs at the further upstream of the DNA. Upon ATP hydrolysis, ADP release reset the conformation, preparing the enzyme for the next movement, and the process can be described as DNA translocation in a rotatory mechanism (Figure 2A).

Analysis of a DNA ligand-bound structure of AfuHel308 has yielded an insight into a possible mechanism for archaea to restart a stalled replication fork by Hjm. The fifth domain of the helicase binds 3’ overhang ssDNA, and the binding could position the enzyme in front of the leading strand at a stalled replication fork, allowing the enzyme to unwind the lagging strand [35]. In the meantime, the binding of the fifth domain to the ssDNA could also function as a molecular brake to regulate the helicase activity [35].

Homologous recombination is another very important cellular process involving distinct types of nucleic acids biomotors. For example, at the very first step of the process, i.e., the end processing of double stranded DNA breaks, two distinct sets of enzymes are involved in the process in the three domains of life: Whereas the main pathway of DNA end processing involves RecBCD enzymes in bacteria [12], the evolutionarily conserved Rad50-Mre11 (MR) complex has been implicated in the same activity in Archaea and Eukarya [18], and it functions together with lineage-specific helicases and nucleases. The archaea-specific enzymes are nuclease NurA and helicase HerA. Biochemical characterization of these enzymes indicates they work in concert to open and process the end at double strand DNA breaks [37]. It has been proposed that Mre11 and Rad50 form a platform of initiation complex and recruit HerA and NurA that are responsible for processing of the end of dsDNAs [38,39].

Although Archaea lack a clear homolog of the bacterial chromosome segregation protein FtsK, but archaeal HerAs and bacterial FtsKs comprises the FtsK-HerA superfamily of translocases together with DNA translocases and DNA packaging ATPases of viral and plasmidic origin [40]. Two types of bacterial nanomotor in this superfamily have been characterized: the bacterial FtsK and RuvB translocases and the phi29 packaging motor. However, while archaeal HerAs can move bidirectionally and implicated in moving by a rotation mechanism [18], the bacterial translocases move unidirectionally, from 5’-3’ direction dsDNA molecules [41-43], and might employ an evolution mechanism as have demonstrated for the phi29 phage packaging machinery [44,45].

The bacterial FtsK motor is comprised of three subdomains: alpha, beta and gamma. Both alpha and beta domains contain a RecA-like nucleotide-binding/hydrolysis fold and form a hexameric ring with a central channel for dsDNA, and ATP binding, hydrolysis and subsequent ADP release result in relative position changes of the two ATPase domains within each subunit, providing the driving force for FtsK translocation along DNA [46]. Further, the motor is given directionality by the regulatory gamma domain, which binds to polarized chromosomal sequences-5'-GGGNAGGG-3', known as KOPS-to ensure that the motor is loaded onto DNA in the specific orientation [47,48]. Since FtsK forms a hexamer ring structure, when the first subunit moves along DNA, the DNA backbone is brought closer to the DNA binding loop of the second subunit. This process facilitates DNA binding at the second unit, which also bring DNA and the third subunit closer and the process repeats, leading to the translocation of the adjacent subunit along DNA. As a result, DNA will be brought around to all the subunits and pumped at the same time.

As for all other members of the superfamily, the packaging machine of the phi29 phage is an AAA+ ATPase. The extensive researches by Guo and co-workers have unambiguously demonstrated that the packaging motor is hexameric protein complex that functions in a one-way revolution model to push the viral DNA into the procapsid [49-53]. The mechanism involves the four electropositive lysine rings of the connector channel that facilitate the DNA revolution, and this occurs without DNA rotation inside the channel during the packaging process [42,54]. Intriguingly, close inspection of the structures of several dsDNA packaging motors has revealed the phi29 packaging mechanism appears to be conserved in several bacteriophages and eukaryotic viruses and cellular dsDNA translocases FtsK and RuvB [43,55]. Such nanobiomotors exhibit strong application potential in nanotechnology and gene therapy [56].

Currently, crystal structure has not been resolved for any HerA helicase yet. However, HerA is a member of the FtsK-HerA helicase superfamily [40], and this evolutionary relationship suggests that HerA could adopt an FtsK-like structure. Indeed, HerA does form a hexameric ring structure as for bacterial FtsK translocase and phi29 packing motor [57]. Possibly, archaeal HerA could have multiple functions such that it functions not only in archaeal DNA repair but also in chromosome segregation during cell division.

## Insights into the motion of Archaeal RadA proteins from structural analyses

More detailed studies on RecA/RadA family of recombinase proteins, including RecA proteins of bacteria, RadAs in archaea, Rad51 and DMC1 proteins in Eukaryotes, have gained important insights into the rotation mechanism for this type of nanobiomotor. These recombinases are essential mediators of homologous recombination, an activity that is required for repairing dsDNA breaks and re-starting of stalled DNA replication forks as discussed above. The RecA/RadA-facilitated strand exchange reaction occurs in two steps: (a) the recombinases bind to ssDNA, forming a nucleoprotein complex, and (b) the nucleoprotein complex invades a homologous dsDNA, such that the invading RadA-ssDNA base pairs with the complimentary strand of the dsDNA whereas the other stranded of the DNA becomes ssDNA, forming a so-called D-loop structure [18]. The D-loop structure is a very important intermediate in DNA repair or DNA replication processes.

### Domain organization of the recombinase proteins

Archaeal RadA and eukaryotic Rad51 proteins show high amino acid sequence identities to each other (> 40%) but they are more distantly related to bacterial RecA proteins, exhibiting ca. 20% sequence identity [58]. Nevertheless, two functional domains are conserved in all the recombinases. While they all contain the core domain including the ATPase motifs, archaeal and eukaryotic recombinases share the N-terminal domain (NTD), whose equivalent is present in the C-terminal domain (CTD) of bacterial RecA recombinases [59]. Noticeably, bacterial recombinases possess a third small domain in their N-termini, which is absent from their archaeal and eukaryotic homologs. Thus, Archaeal and eukaryotic recombinases are also more closely related to each other at protein domain structure.

X-ray crystallographic studies of RecA family proteins indicate that the core domain consists of twisted β-sheet, flanked by α-helices on both sides. Such structure in RecA coordinates ATP binding together with the β-sheet at the carboxyl end and an α-helix at the amino terminus (Walker A motif) [60]. Also included in the core domain are L1 and L2, two disordered regions (or loops) that interact with ssDNA [61]. The NTD domain of RadA and Rad51 comprises of four helices, including a helix-hairpin-helix motif to bind to a dsDNA. Its functional homolog in bacterial RecAs is the CTD domain albeit it adopts a different structure. Further, structural analysis has revealed a link region between the two domains in RadA/Rad51, which is responsible for protomer polymerization (polymerization motif, PM). Interestingly, this domain is absent from bacterial RecAs. The bacterial recombinases employ the small N-terminal domain for protomer polymerization [60].

### Formation of RadA-ssDNA filaments and their stabilization

While the structure of the *E. coli* RecA was resolved in 1992 [60], little progress was made in the understanding of RecA family recombinases in the following 10 years. Then, different quaternary structures have been obtained for bacterial RecA proteins including the structures associated with ADP or in the absence of any nucleotide and the RecA-ssDNA filaments with a compact helical pitch, which is enlarged in the presence of ATP or its analogs [62]. Electron microscopic studies of the active filament indicate that ATP binding results in a reorientation between the CTD and the ATPase domain of RecA and a rigid body rotation between two subunits, which then leads to the formation of different interface between subunits [62].

**Figure 3:**
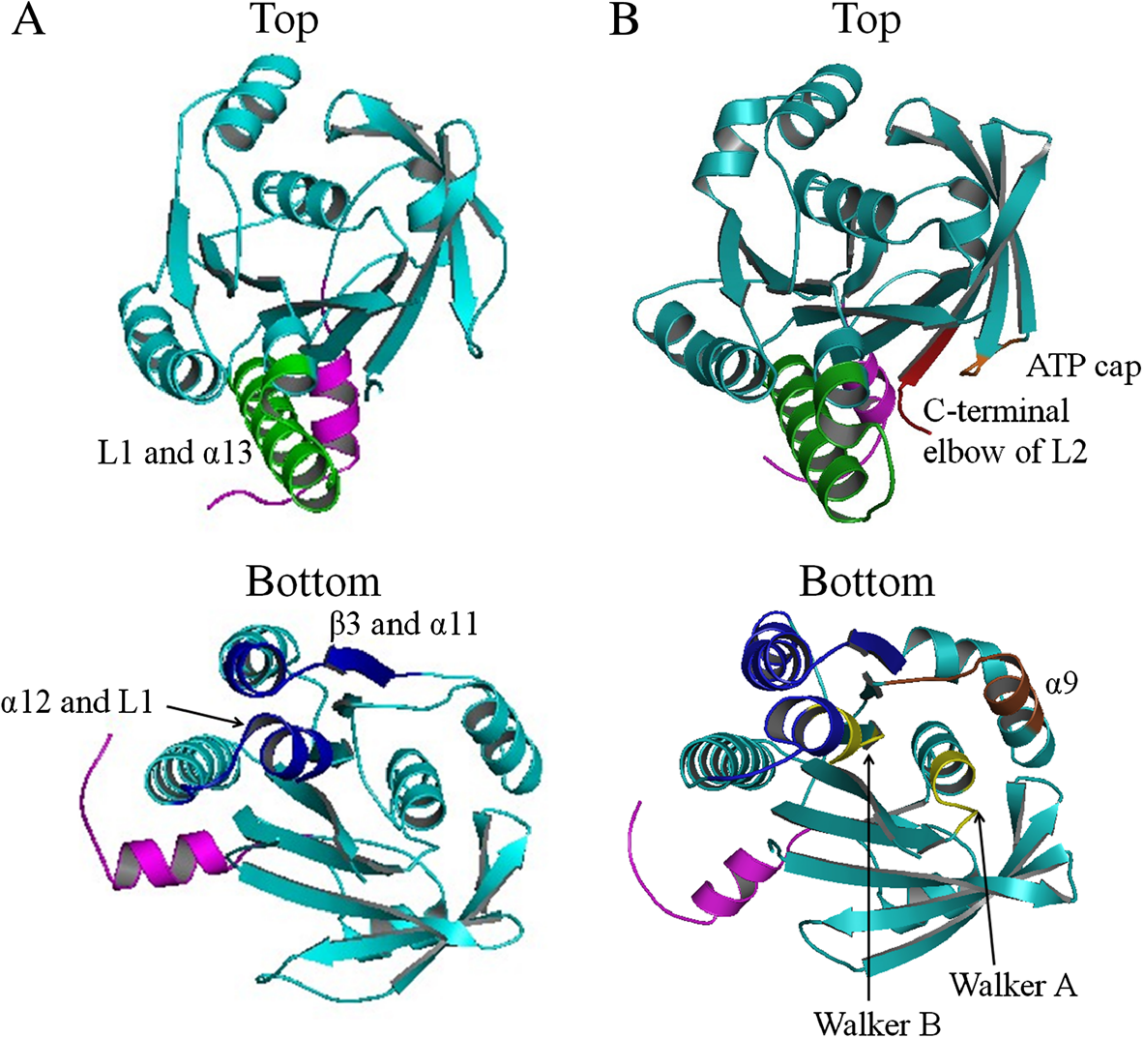
**Subdomain interactions at the interface of two neighboring ATPase domains in the PfuRadA ring structure (PDB: 1PZN) (A) and in the MvRadA filament structure (PDB: 1T4G) (B).** In the ring structure, the interface of two neighboring ATPase domains is stabilized by L1 and α13 (in green) from one subunit (top) and α12, L1 and β3/α11 turn (in blue) from the adjacent subunit (bottom), while in the MvRadA filament structure, additional subdomains that interact at the interface between two adjacent ATPase core domains include ATP cap (in orange), the C-terminal elbow of L2 (in red) from one subunit (top), and Walker A, B motifs (in yellow), a part of α9 (in brown) from another subunit (bottom). The NTD is not shown for charity and the linker region between NTD and the core domain, including PM and SRM, is shown in pink.

RadA proteins also form different quaternary structures under different conditions. They form a ring structure in the absence of nucleotide, a form implicated in RadA storage in order to decrease ATP pool consuming [63] while the extended right-handed filament with AMP-PNP is suitable for ssDNA-binding and forming nucleoprotein filament [61]. Comparative studies of the ring and right-handed filament structures of RadAs have revealed interesting features in their structures [61]. In the absence of ATP, the Walker motifs adopt a conformation to hold the ATP-binding site open and do not interact with the adjacent ATPase domain [63,64]. The inter-subunit surface is only formed by L1 and α13 from one monomer and α12, L1 and the β3/α11 turn from the adjacent monomer [63]. In the crystal structure of right-handed filament, two adjacent protomers are assembled in the head-to-tail fashion by a nucleotide such as an ATP analog, which interacts with the Walker A and Walker B motifs of one protomer and with another region called ATP cap of the adjacent protomer (Figure 3) [61]. When ATP is buried in the amino acid residues of two neighboring subunits, the interaction surface is largely increased. In the right-handed filament formed by *Methanococcus voltae* RadA (MvRadA), about 2550 Å buried surface is observed between two adjacent monomers, much larger than that observed in the ring structure. The additional interaction surface causes a ~30° rotation between two neighboring ATPase core domains [61].

Additional conformational changes induced by ATP-binding include the changes and re-arrangement of ssDNA-binding loops, L1 and L2. The bound ATP interacts directly with the N-terminal elbow region of L2 and stabilizes its conformation. Since the γ-phosphate of ATP interacts with L2, ATP hydrolysis should yield an extensive allosteric regulation on the structure of L2 region, as have shown with an archaeal RadA-ADP structure [65]. This is consistent with the biochemical analysis in which ATP and ADP function in regulating the DNA-binding activities of RadA [66]. Apart from the direct interaction between L2 and the ATP analog, conformational changes in the core domain include repositioning of L1 and L2. For example, only L1 hairpin is located near the cavity of an archaeal RadA ring in the absence of any nucleotide [63]. When forming right-handed filaments in the presence of an ATP analog, L2 is positioned close to the filament axis along with L1 due to the interaction between L2 in one protomer and the γ-phosphate group of the ATP analog that is bound to the adjacent protomer [61]. The arrangement of L1 and L2 along the filament axis is favorable for ssDNA binding. Alternatively, the binding of ssDNA may also fix the alignment of L1 and L2, thus stabilizing the right-handed filament structure of RadA.

All RecA family recombinases require metal ions for their activity. Several metal ions have been co-crystallized with these proteins, including Mg^++^, Ca^++^, NH_4_^+^ and K^+^. Mg^++^ is stabilized at the ATP binding sites in several recombinases [61,67]. For the RecA-ssDNA/dsDNA filament, the Walker motifs coordinate Mg^++^ and the α- and γ-phosphate of ATP. Interestingly, the amino acid residues responsible for the coordination are well conserved in the RecA family proteins [61] and they interact with diverse metal ions. In fact, when Mg^++^ is replaced with Ca^++^, a human Rad51 exhibits an enhanced strand exchange activity [68]. Likewise, Ca^++^ also stimulates the strand exchange activity of Human and Yeast DMC1 as well as RadA proteins, promoting the formation of nucleoprotein complexes in the presence of ssDNA [69-71]. Ca^++^ is not the only metal ion that stimulates the process. For example, Human and Yeast Rad51 and DMC1 also exhibit higher activities in the presence of KCl and (NH_4_)_2_SO_4_. More interesting, similar effects of Ca^++^ and K^+^ on RadA have recently been reported [72,73]. A more detailed analysis has been conducted with the archaeal MvRadA filament. In the presence of K^+^, two K^+^ ions interact with the γ-phosphate, suggesting that K^+^ could regulate the ATPase activity. Another noticeable difference in the structures is that two K^+^ ions bridge the interaction between the L2 region and the γ-phosphate and stabilize the conformation of the L2 loop region. This suggests that K^+^ plays a role both in both mediating ATPase activity and facilitating RadA-ssDNA nucleoprotein formation [73]. Interestingly, the structural analysis of MvRadA in the presence of Ca^++^ has revealed that Ca^++^ could also stabilize L2 just as K^+^[72]. Further investigations with more metal ions and diverse recombinases should yield more insights into the effects of metal ions on the recombinase filament formation.

### The clockwise axial model of RadA

Two new forms of RadA-ssDNA complex have been revealed recently, the overwound right-handed filament and the left-handed filament of *S. solfataricus* RadA. In the overwound right-handed filament there is an additional ~60° rotation between two adjacent protomers comparing to the right-handed filament [64] and a further ~120° axial rotation is required to yield the left-handed filament [74]. These conformations are useful in interpreting additional rotations from the right-handed filament such that Wang et al. have proposed a model for the strand exchange by these recombinases, namely the clockwise axial model of RadA [75]. In the proposed model, the first step is that ATP binding to RadA induces conformational change in RadA to initiate the rotation, yielding a presynaptic complex, and this is based on the fact that RadA proteins show a ring structure in the absence of ATP but form a right-handed filament with ssDNA when in the presence of ATP. Then, ATP hydrolysis in the presynaptic complex leads to a conformation status that fits to the structure of the overwound right-handed filament and the consequence of this conformation change is to force strand exchange to form a D-loop structure. Next, the complexes release ADP and dissociate from the DNA strands yielding a conformation of RadA resembles those in the left-handed filament (reviewed in Wang et al., 2008 [75]).

**Figure 4:**
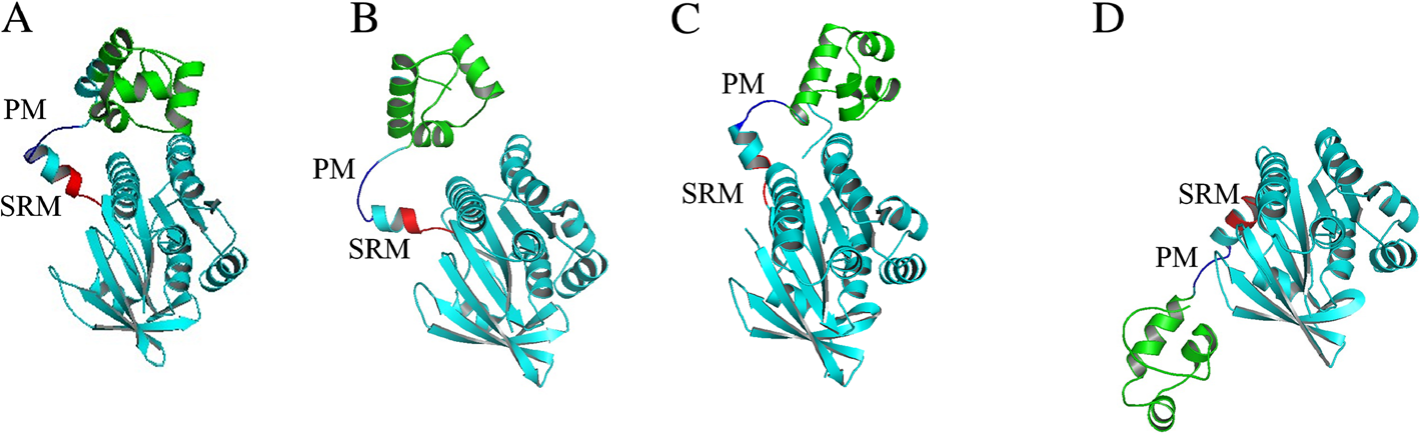
**The reorientation of NTD (in green) to the core domain in different RadA assemblies induced by conformational changes of SRM (in red) and PM (in blue).** The core domains from four different assemblies are fixed to show the reorientation of NTD and conformational changes of SRM. **A**: Protomer in the ring structure of PfuRadA (PDB: 1PZN), **B**: Protomer in the right-handed filament of MvRadA (PDB: 1T4G). **C**: Protomer in the overwound right-handed filament of SsoRadA (PDB: 2BKE), **D**: Protomer in the left-handed filament of SsoRadA (PDB: 2DFL).

Further insights into the rotation by RadA recombinases during strand invasion have been gained from identification of a subunit rotation motif (SRM) located between the polymerization motif (PM) and the core domain [74]. The SRM domain adopts different conformations in the four structures of RadA nucleoprotein filaments discussed above (Figure 4). Since the interaction of PM with the core domain from the adjacent subunit remains the same, reorientation between PM and the core domain produces conformational changes in SRM, resulting in rotation between two neighboring protomers. In the MvRadA right-handed filament, domain L1, L2, and the HhH motif of the N-terminus face the filament axis. Since L1 and L2 are implicated in the ssDNA binding while the HhH motif is responsible for dsDNA binding, this reveals a mechanism for the nucleoprotein complex to interact with dsDNA [61]. When the right-handed RadA-ssDNA switches to the overwound right-handed filament, the rotation brings L1 and NTD together to form a palm structure at the outer surface of the filament. This structure makes the DNA-binding pocket that can accommodate homologous pairing between the invading strand and the complimentary strand in the template DNA [76]. The left-handed filament reflects the last step of clockwise axial rotation in which the DNA-binding motifs are located at the outermost surface. This is a structure that favors extrusion of ssDNA after homolog pairing (reviewed in Wang et al., 2008 [75]).

The conformational changes of SRM could be coupled to ATP binding, hydrolysis and DNA binding. In the right-handed filament of MvRadA with AMP-PNP, the guanidinium group of a conserved arginine residue in SRM (Arg83 for SsoRadA, Arg74 for MvRadA, referred to R0) forms two salt bridges with a glutamate residue from the same subunit (Glu105 for SSoRadA, Glu96 for MvRadA, referred to E1) and a glutamate residue from the adjacent subunit (Glu156 for SSoRadA, Glu157 for MvRadA, referred to E2). The E1-R0-E2 triad acts to stabilize the binding of AMP-PNP between the two neighboring core domains. Whereas L1 and L2 are aligned to the filament axis, E1 and E2 are excluded from that in order to avoid the repulsion between the acidic groups of the two glutamates and the phosphate groups in ssDNA. After ATP hydrolysis, the ATP binding site is open up and the two glutamates also move away from R0 as have observed in the left-handed RadA-ssDNA filament. In this conformation, the exposure E1 and E2 residues may interfere DNA binding because they are located on the path of L1 and L2. The alignment of L1, L2, E1 and E2 residues provides the molecular basis for extruding ssDNA and consequently stabilizing the heteroduplex DNA after homologous pairing [75]. Nevertheless, more RadA-DNA complexes need to be obtained and analyzed to reveal more detailed molecular mechanisms for the strand invasion in homologous recombination.

## Other archaeal AAA + ATPases involved in DNA repair

RadA paralogs represent another major group of AAA + ATPases involved in DNA damage repair in Archaea. These proteins contain an ATP-binding domain but lack the dsDNA-binding domain of RecA family recombinases [17]. The exact function of these ATPases is still obscure although they have been implicated in regulating homologous recombination in archaea.

RadB is a RadA paralog widely conserved throughout Euryarchaeota, one of the major branches of the Archaeal Domain [77]. X-ray crystal studies of a euryarchaeal RadB show that it is a dimer, adopting a helical structure [78]. In this structure, the ATP-binding sites are deeply buried in the two molecules. As a result, the enzyme exhibits only a weak ATPase activity of these enzymes [78]. Therefore, dimer could be considered as a form that is unfavorable for polymerization. Interestingly, ATP stimulates the DNA-binding activity of the RadB enzyme, suggesting that the stimulation effect of ATP must have achieved from inducing conformational changes of RadB [77]. More protein crystallographic analysis needs to be conducted for RadB proteins in order to test the hypothesis.

The other widespread RecA family proteins are known as “aRadC” (archaea RadC) [17]. Although also called RadA paralog initially, these proteins reliably form a distinct clade by themselves, which is distinct from the RadA and their homologs. Characterization of RadC proteins of *Sulfolobus* has yielded some insight into their possible functions. Biochemical studies showed that one of RadC members in *S. tokodaii*, RadC1 (Sto0579), enhances the ATPase and strand invasion activities of RadA [79], while another RadC member, RadC2 (Sto1830), interacts with both RadA and Hjc, a Holliday junction resolvase [80]. In vivo functional analyses have shown that two archaeal homologs, RadC1 and RadC2, in *S. islandicus* are involved in DNA repair [81], though the exact role of RadC1 in HR is still under debate [66,79,82]. Crystal structures of several RadC proteins have been resolved [82,83]. RadC proteins form a hexamer ring structure with ADP and the ADP is located at the interface of monomers, a structure characteristic for many known biomotors. Gel filtration analysis further confirms the roles of nucleotides in mediating assembly of StoRadC1. More interestingly, dimer is observed when the nucleotide is not sufficient, suggesting similar assembly process to that of RadB (unpublished data). Moreover, StoRadC1 proteins also possess DNA-stimulated ATPase activity [79,82] and pre-incubated with ATP or ADP dramatically stimulates the DNA-binding activity of StoRadC1 (unpublished data), indicating the coupling cooperation of RadC-DNA and RadC-ATP interactions. Therefore, RadC provides another interesting model for studying interactions of motor proteins of DNA repair systems.

## Conclusions and perspectives

Studies of the biomotors of archaeal DNA repair machineries indicate that the mechanisms for them to perform their functions are diverse. SF2 helicases translocate along DNA in a classic fashion of rotation. For the members of Ftsk-HerA superfamily, the phage phi29 packaging motor, each subunit moves along DNA during viral DNA packaging and the sequential translocation in each subunit of the hexameric protein lead to the rotation of DNA binding site around the protein ring, a process described as the evolution movement. FtsK cellular nanobiomotors could function under the same principle. Since the mechanism of DNA unwinding by archaeal HerAs remains to be demonstrated, it is intriguing to hypothesize that these archaeal nanomotors could also adopt a revolution mechanism to unwind DNA because they fall into the same protein superfamily with bacterial FtsK proteins.

RadA provides a prototype nanobiomotor for studying conversion of chemical energy to mechanical movement. In the clockwise rotation model, ATP binding and hydrolysis at the core domain result in repositioning of NTD and core domain and rotation between two adjacent subunits. Unlike most nanobiomotors that translocate along DNA, RadA polymers do not move along ssDNA or dsDNA but rotate around the axis like F1 ATPase. It is believed that such rotation could allow multiple conformational changes at their DNA binding sites, regulating DNA affinity and the reposition of ssDNA and dsDNA during the strand exchange reaction. To date, this hypothesis needs to be tested with more crystal structural analyses of RadA/Rad51/RecA proteins.

Recently, all macromolecules have been tested as vehicle for drug delivery, including lipids, and DNA, RNA and protein nanoparticles. Important progresses have been made for all of them. Amongst the proteins that could be to be exploited for drug delivery, nanobiomotors are important examples. Two types of nanobiomotors are well known: linear and rotary motors [52]. Linear motor proteins, such as kinesin, dynein, and certain myosins, step unidirectionally along linear tracks as for microtubules and actin filaments. Rotary biomotors, such as DNA repair helicases, unwind dsDNA molecules for damage repair. The third type nanobiomotor, represented by the phage phi29 packaging motor, moves via revolution as the Earth travels around the Sun [51,53]. It has been reasoned that several cellular nanobiomotors function under this principle, including FtsK, a member of the HerA/FtsK superfamily.

To date, amongst different types of nanoparticle studied for drug delivery the research on the application of lipid nanoparticle in delivering short interference RNAs (siRNAs) is most advanced [1]. However, the usefulness of the nanomatereials has been compromised by the finding that multiple cellular signaling effectors are required for the initial cellular entry of lipid nanoparticles during which the internalized siRNAs undergo exocytosis [2]. Nanobiomotors are primary carriers for drug delivery. For example, DNA and RNA nanoparticles have been developed alternative systems for siRNA delivery. It has been found that a DNA tetrahedron was taken up by mammalian cells and remained substantially intact within the cells for at least 48 h after transfection [84]. This DNA cage was used to deliver siRNA into mammalian cells [85]. Furthermore, one such system uses a hollow DNA box containing proteins that induce apoptosis, or cell death, that will only open when in proximity to a cancer cell [86,87]. Important progresses have been made in developing RNA nanoparticles for delivering siRNAs and this initiative has been strongly inspired by the hexameric RNA ring of phi29 packing machine. Many polymers of RNA molecules have been synthesized and these RNA structures show extremely high stability and are implicated in using as a vehicle for drug delivery. Preliminary finding that RNA nanoparticles can specifically target to cancer for the delivery of siRNA, miRNA, and ribozymes without accumulation in liver, lung and other normal organs without toxicity, making RNA nanoparticles ideal reagents for cancer treatment [88,89].

Once the structures of biomolecular motors have been revealed with the involved processes understood, these principles of cellular movements can be used to build up various nanobots to be used in drug delivery. Due to the evolutionary conservation of information-processing machineries between Archaea and Eukarya, archaeal and eukaryotic nucleic acids motors are also more closely related to each other than to bacterial counterparts. Above all, many archaea are hyperthermophiles and the encode proteins that are more stable than the proteins derived from other sources. This is one of the reasons that thermophilic proteins are more widely used in crystallographic analysis of protein structure. For the same reason, when developed as nanobiomaterials, these archaeal proteins including archaeal nanobiomotors would be proven to be invaluable in delivery of biomedicine in molecular therapy of cancer treatment. Currently, archaeal nanobiomotors still represent a largely unexplored world for nanotechnological research. Once started, we anticipate very rapid research progresses in the field and the application of these exceptionally stable nanobiomotors in biomedicine and biotechnology in a near future as for DNA and RNA nanotechnologies.

## Competing interests

The authors declare that they have no competing interests.

## Authors’ contributions

WH, YS, and QS contributed the first draft and WH prepared the figures. QS revised the article. All authors read and approved the final manuscript.
